# Health professionals’ preferences with the use of pegfilgrastim on-body injector at oncology centers in 8 cities in Colombia

**DOI:** 10.1186/s12913-023-09454-z

**Published:** 2023-05-23

**Authors:** María Alejandra Larrarte-González, Mariana Pineda-Posada, Álvaro Andrés Gaitán, Jenny Amaya-Amaya, Kelman Ojeda

**Affiliations:** 1 Centro de Atención e Investigación Médica CAIMED, Chía, Colombia; 2 Amgen Biotecnológica SAS, Carrera 7 #123-35, piso 6, Bogotá, Colombia; 3 Amgen Inc., Thousand Oaks, USA; 4 Centro Javeriano de Oncología of the Hospital Universitario San Ignacio, Carrera 7# 40-62, Bogotá, Colombia

**Keywords:** Pegfilgrastim, Granulocyte colony-stimulating factor, Chemotherapy-Induced Febrile Neutropenia, Colombia, Cancer Care Facilities

## Abstract

**Background:**

Febrile neutropenia associated with some chemotherapy regimens can lead to potentially fatal complications and high health care costs. Administration of pegfilgrastim using an On-Body Injector (OBI) may be more convenient for cancer patients and physicians in countries with limited access to high-complexity healthcare. This study aims to describe physician and nurse preferences regarding different options for administration of pegfilgrastim at cancer centers, the chemotherapy schemes for which pegfilgrastim is most frequently prescribed and how healthcare providers prioritize certain administration schemes according to patients’ access to healthcare services.

**Methods:**

Observational, descriptive, cross-sectional study and survey, conducted between 2019 and 2020, to describe physician and nurse preferences regarding options for administration of pegfilgrastim at cancer centers, the demographics of the study population and characteristics of participating cancer centers. It included 60 healthcare professionals practicing at oncology centers from 8 cities in Colombia who were contacted and surveyed via telephone. Quantitative continuous variables were summarized using central tendency and dispersion measures.

**Results:**

It was found that 35% of participants are haemato-oncologists, oncologists or hematologists, 30% are general practitioners, and 35% are other healthcare professionals (i.e., nurse, oncology nurse and head nurse). Our study shows that 48% of physicians prefer the use of OBI, particularly in the scheme of 24 h after myelosuppressive chemotherapy administrations. Regardless of patient frailty and travel time to the clinic, over 90% of healthcare providers (HCPs) prefer to prioritize preventing the patient from having to return to the clinic for pegfilgrastim administration as well as to increase healthcare staff availability through the use of OBI.

**Conclusions:**

The present study is the first one in Colombia that sought the reasons behind HCPs’ choice to use OBI pegfilgrastim. Our results indicate that most professionals prefer to avoid the patient having to re-enter the care center for pegfilgrastim administration to facilitate access to healthcare for patients; patient characteristics and ease of transport are determining factors for respondents when choosing an option for drug administration. We found OBI is the preferred alternative by most HCPs and a good resource optimization strategy in the context of cancer patients’ health care in Colombia.

**Supplementary Information:**

The online version contains supplementary material available at 10.1186/s12913-023-09454-z.

## Background

Febrile neutropenia (FN) is an adverse event associated with some chemotherapy regimens that can lead to fatal complications [1, 2]; additionally, it is associated with high healthcare costs and treatment delays [3]. The incidence of FN may be as high as 117 cases per 1,000 cancer patients [1]. In patients with non-myeloid disease receiving myeloid-suppressive therapy, and whose risk of FN is ≥ 20% and 10–20% with an additional risk factor, primary prophylaxis with granulocyte colony-stimulating factors (GCSF) is recommended [1, 2]. Pegfilgrastim is a sustained-duration GCSF which, due to its single-dose scheme and favorable efficacy and safety profile, is the most widely used and distributed medication of its kind worldwide [1, 3]. Pegfilgrastim should be administered one day after chemotherapy. Administration within the first 24 h is associated with an increase in myeloid progenitor cells susceptible to toxicity; likewise, administration after 72 h increases the risk of FN due to fewer receptor cells in the bone marrow [2]. On-body injector (OBI) was created to ensure that pegfilgrastim is administered one day after chemotherapy without the patient having to return to the hospital. This is a battery-operated electromechanical device that is applied to the patient’s skin on the day of chemotherapy and injects the drug 27 h after chemotherapy administration. OBI has been reported to achieve a significant risk reduction for the occurrence of febrile neutropenia [3, 4]. Previous studies about OBI use have reported an improvement in the workflow at oncology centers [4] and in patient satisfaction because it does not require the patient to re-enter the center for medication administration [4–6]. Furthermore, a recent study evaluated the effects of OBI use on compliance and persistence to GCSF; it found approximately 30% more persistence to GCSF for patients using OBI, when compared to patients using another method of pegfilgrastim administration or no GCSF at all [7].

The use of OBI could provide an alternative for timely pegfilgrastim administration in countries with barriers to healthcare for cancer patients. Previous studies have analyzed such difficulties in Colombia, emphasizing on the economic limitations imposed by the expenses of transportation, lodging, food or secondary payments for medical services. Geographical obstacles are related to the absence of health services in rural areas, particularly for complex services such as clinical laboratories, specialized cancer centers or diagnostic imaging equipment, which are located mainly in urban areas [8]. In addition to these obstacles, it is important to consider that a great proportion of cancer patients have frailty as a secondary condition related to their primary disease, which may limit their ability to reach healthcare facilities in certain cases [9]. This must also be considered as a part of cancer patients’ welfare in their treatment.

Obstacles to healthcare access are not only present but they are not actively measured in most cases, which limits the opportunities to intervene them and reduce the impact they have on health outcomes for patients. One study measured access to cancer care in Colombia and reported that delays in diagnosis and treatment administration impacts survival and is associated with worse health outcomes for cancer patients in Colombia [10].

A study that included more than 40 centers in Germany, reported that patients showed a slight preference for the use of OBI for the administration of pegfilgrastim; however, the authors suggested that the high availability of pre-filled syringes (PS) in several locations across Germany could influence healthcare providers (HCPs) to choose PS over OBI (prescribing OBI could increase the cost of treatment) [11]. Another study evaluated HCPs’ choices from a center in the United States when administering pegfilgrastim and found that they usually prefer to use administration options they have prescribed in the past. The authors analyzed patients’ perspective as well and found that patients prefer the options they are most familiar with; however, most patients did report a perceived burden when having to return to the clinic for pegfilgrastim administration after having received myelosuppressive chemotherapy [5]. This is consistent with similar results reported in other studies [12].

The advantages of OBI have been studied mostly in the context of countries with the possibility of ensuring access to complex healthcare for cancer patients; however, this is not the case for third-world countries where the population faces economic and administrative burdens. Additionally, for those countries, HCPs are usually located in the main cities where patients are expected to travel from rural areas. It is important to evaluate how HCPs perceive the use of OBI in Latin American countries, where they must consider specific barriers that patients might face when accessing healthcare and how this may affect health outcomes.

Considering the relevance of timely pegfilgrastim administration, this study aims to describe the preferences of physicians and nurses regarding pegfilgrastim administration options in community oncology centers in Colombia. Additionally, this study seeks to describe the characteristics of participating oncology centers, demographics of the study population, the conditions for which pegfilgrastim is usually prescribed and the risk scores for neutropenia most frequently used at participating centers.

## Methods

This is an observational, cross-sectional descriptive study using a survey. The main outcome was defined as the preferences of physicians and nurses regarding pegfilgrastim administration options in cancer centers, presented as the percentages of professionals who prefer the use of OBI or pre-filled syringes (Pre-FS) (See Appendix 1 for survey structure). Convenience sampling was used for this study. Demographic characteristics of respondents, characteristics of the cancer centers, years of professional experience, percentages of professionals who prefer the use of specific risk scores of FN, and more frequent conditions for which pegfilgrastim is prescribed are included as secondary outcomes.

This study used the methodology proposed by Hauber et al. [5] as the key methodological reference with the objective of standardizing the questions related to the use of OBI, according to the reports of previous studies. The survey used by Hauber et al. was qualitatively pretested with convenience samples of five oncologists and/or hematologist/ oncologists and five patients with self-reported breast cancer, non-Hodgkin´s Lymphoma, non-Small Cell Lung Cancer, or colorectal cancer who had received prophylactic administration of pegfilgrastim [5]. Our study is based on the survey applied to physicians and the data was collected by an electronic case reporting form. The survey was not applied to patients in this study. The survey structure included information about different options to administrate pegfilgrastim, followed by questions exploring participants’ preferences on this regard. Table 1 presents the administration alternatives introduced in the survey and the key elements related to each of them. To rank the importance that physicians and nurses give to different reasons for choosing one option to administer pegfilgrastim over another, participants were asked to assign points to each of those reasons from a total budget of 25 points, so that each reason was evaluated from 0 to 25 points, with the highest number of points awarded to the preferred characteristic. The total sum of the scores for all characteristics per option should be 25 points.

**Table 1:** Pegfilgrastim administration options and their features*

Options for pegfilgrastim administration	Potential characteristics
Administration on the same day of myelosuppressive chemotherapy within a clinic, by a health professional.	▪ Not having to return to the clinic 24 h after chemotherapy to receive the injection ▪ Not having to return to the clinic 48–72 h after chemotherapy to receive the injection. ▪ No need to place the body injector for Neulastim®.
Administered when the patient returns to the clinic 24 h after myelosuppressive chemotherapy, specifically for the purpose of receiving an injection of pegfilgrastim, by a healthcare provider.	▪ Not having to return to the clinic 48–72 h after chemotherapy to receive the injection. ▪ No need to place the body injector for Neulastim®.
Administered when the patient returns to the clinic 48–72 h after myelosuppressive chemotherapy, specifically for receiving an injection of pegfilgrastim, by a healthcare provider.	▪ Not having to return to the clinic 24 h after chemotherapy to receive the injection ▪ No need to place the body injector for Neulastim®.
Administration at home using the Onpro Neulastim®™ kit (OBI) that has been attached to the patient’s arm or abdomen at the clinic; approximately 27 h (a little more than 1 day) after myelosuppressive chemotherapy.	▪ Not having to return to the clinic 24 h after chemotherapy to receive the injection ▪ Not having to return to the clinic 48–72 h after chemotherapy to receive the injection.

*Adapted from (1)

Furthermore, the survey included three hypothetical patient profiles for which participants were asked to choose their preferred pegfilgrastim administration alternative. The first one corresponded to a typical patient requiring pegfilgrastim use which aimed to evaluate the preference as an HCP for the use of pegfilgrastim, the second and third scenarios were specific and required professionals to make a choice taking into account other variables. Clinical cases were presented as follows:

### Clinical case 1

This scenario asked HCPs to choose a pegfilgrastim administration method based solely on their preference as an HCP regardless of any particular patient.

### Clinical case 2

A 67-year-old woman with chronic obstructive pulmonary disease (COPD) who presented with a second recurrence of breast cancer involving bone, lung and liver, and an Eastern Cooperative Oncology Group (ECOG) performance status of 2. She is starting a two-drug chemotherapy regimen due to rapidly progressing visceral metastases. She traveled 2 h to the clinic to receive her chemotherapy infusions.

### Clinical case 3

A 29-year-old woman with early-stage breast cancer who was having a first course of dose-dense adjuvant chemotherapy after undergoing limited surgical resection of the primary breast tumor. She had no significant medical history or concomitant illnesses and had an ECOG performance status of 0. She traveled 30 min to the clinic to receive her chemotherapy infusions.

The survey structure as it was used by the investigators can be found in Appendix 1 (Flowchart 1 to 4). Data were collected through telephone surveys performed from December 2019 to April 2020.

Inclusion criteria were defined as: 1. Physicians (oncologists, haemato-oncologist, hematologist, or general practitioners) who prescribe myelosuppressive chemotherapy, have prescribed pegfilgrastim to mitigate FN within the past 6 months, and have had experience with administrations of pegfilgrastim by Pre-FS and OBI or both. Nurses (nurse/oncology nurses/head nurse) who have had management or experience applying Pre-FS and OBI to mitigate FN in the last 6 months, as prescribed by a physician. The exclusion criterion was defined as doctors and nurses who have no experience in the use or prescription of OBI.

### Statistical methods

A descriptive analysis was carried out using the statistical software Stata14. Quantitative continuous variables were summarized using central tendency and dispersion measures (means, median, standard deviation and ranges according to their distribution, as appropriate). The qualitative variables, both nominal and ordinal, were described through absolute and relative frequencies.

## Results

**Fig. 1:**
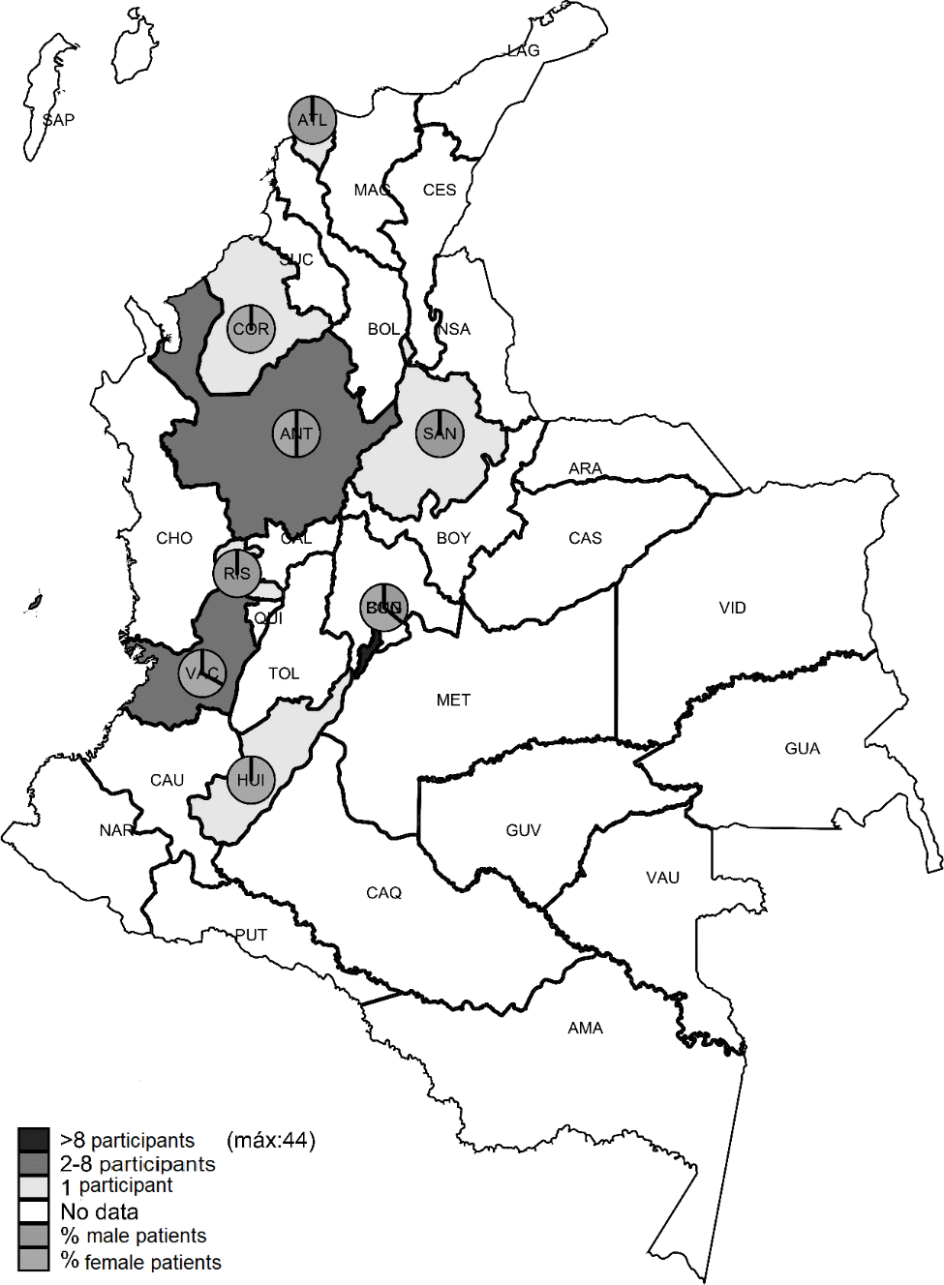
Distribution of participants among Colombian territory Heat map-representation of the spatial distribution of participants among Colombia’s departments, with a distinction between female and male participants. Initials for each department’s and cities’ names are shown on the map

Information and distribution by department from 60 participants from 8 cities (Bogotá, Medellín, Cali, Barranquilla, Bucaramanga, Monteria, Neiva and Pereira) are shown in Fig. 1. The cities with the highest participation were Bogotá, Medellín and Cali, with 73.33, 13.33 and 5.0%, respectively.

Socio-demographic characteristics are presented in Table 2. In Colombia, there are three main ways to access the health system: subsidized regime (government payment), contributory regime (workers), and private insurance. In this study, 83.3% HCPs provided their services to the last two groups. It was found that 35% of participants are haemato-oncologists, oncologists or hematologists, 30% are general practitioners, 23.3% are oncology nurses and 11.7% are head nurses. The average time of professional experience of surveyed participants was 10.13 years (SD = 5.53 years) at the time of the survey.

**Table 2:** Study population characteristics

Variable	Participants	
	n = 60	
	n	%
**Age in years** ^**a**^	37,8 (5,54)	37,5 (34,87 − 41,78)
**Age group**		
20–30 years old	5	8,3
30–40 years old	36	60
40–50 years old	19	31,7
**Gender**	279	
Female	36	60
Male	24	40
**City where participants work**		
Bogotá	44	73,33
Medellín	8	13,33
Cali	3	5,00
Barranquilla	1	1,67
Bucaramanga	1	1,67
Montería	1	1,67
Neiva	1	1,67
Pereira	1	1,67
**Professional degree**		
Nurse/ oncology nurse	14	23,33
Head nurse	7	11,67
Haemato-oncologist	6	10,00
Hematologists	6	10,00
General Practitioner	18	30,00
Oncologists	9	15,00
**Years of professional experience** ^**a**^	10,13 (5,53)	9 (5,5–14)
**Medical practice facility**		
Oncology Reference Center/Hospital	32	53,33
Other Hospital / Academic Clinic	19	31,67
Clinic/ Non-academic hospital	10	16,67
Private Practice Group	3	5,00
**Facility in which patients receive immunotherapy**		
Both inpatient and outpatient cases	40	66,67
Outpatient / non-hospital consultation	2	3,33
Hospital Outpatient consultation	3	5,00
Inpatient	3	5,00
Inpatient or ambulatory consultation	12	20,00

^**a Values reported in terms of mean (standard deviation) and median (p25-p75)**^

No clear preference was found in professionals regarding administration of chemotherapy at a specific setting, with 66.67% (n = 40) of them attending both inpatient and outpatient cases. When analyzing the preferences by profession, we found that oncology nurses prefer OBI in a greater proportion than other professionals with 92.3% (n = 12), reporting OBI as their first choice. From the group of hematologists who participated, 66% (n = 4) of them reported a preference for OBI. 80% (n = 4) of general physicians and 57% (n = 4) of head nurses prefer OBI over other methods of administration. Interestingly, among the group of hemato-oncologists only 33% (n = 1) preferred OBI.

**Fig. 2:**
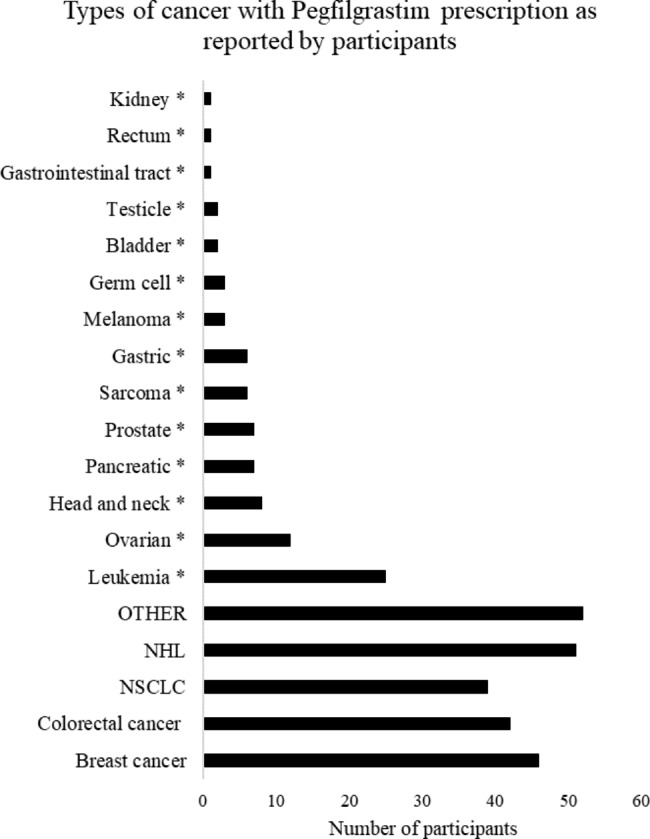
Types of cancer with Pegfilgrastim prescription as reported by participants Frecuency of each type of cancer reportedly treated with Pegfilgrastim according to healthcare professionals that participated in the survey. Breast cancer, colorectal cancer and Non-Hodgkin’s lymphoma are the most frequent diagnoses among patients treated with Pegfilgrastim

When analyzing the characteristics of the patients treated at these cancer centers, a median of 150 patients were treated in a month, of which 66% received chemotherapy, as presented in Table 3. When evaluating the indications and uses of pegfilgrastim, most of the professionals (85%) reported they use the medication in cases of non-Hodgkin Lymphoma (NHL), followed by breast cancer (76.6%), colorectal cancer (70%) and lung cancer (65%). Of all participants, 86.6% reported additional indications outside the group of conditions already mentioned and were outlined by HCPs as well, for example: it was found that 45.6% of participants had treated leukemia patients with pegfilgrastim, followed by 24.56% (n = 14) in the case of ovarian cancer. These results are represented in Fig. 2.

**Table 3:** Characteristics of cancer patients treated and pegfilgrastim administration options at oncology centers

Variable	Participants	
	n = 60	
	n	%
**Number of patients with breast cancer, NHL, NSCLC and/or colorectal cancer seen in one-month** ^**a**^	150 (50–300)	
**Percentage of patients with breast cancer, NHL, NSCLC and/or colorectal cancer with pegfilgrastim** ^**b**^	60,3 (25,6)	70 (40–80)
***Options for pegfilgrastim administration that have been used by physicians***		
**Same day as myelosuppressive chemotherapy, in the clinic, by a HCP**	40	66,7
**Administered when the patient returns to the clinic, 24 h after myelosuppressive chemotherapy, specifically to receive an injection of pegfilgrastim from a HCP**	48	80
**Administered when the patient returns to the clinic, 48–72 h after myelosuppressive chemotherapy, specifically to receive an injection of pegfilgrastim from a HCP**	25	41,7
**Home administration using Neulastim (OBI) that has been attached to the patient’s arm or abdomen in the clinic; approximately 24 h (just over 1 day) after myelosuppressive chemotherapy**	47	78,3

Preferences regarding the time and means of administration of pegfilgrastim by professionals indicate that 48.02% of them give priority to the use of OBI, treating patients at home through this device; whereas Pre-FS were applied 34.83% in office / clinic / outpatient infusion center and 17.8% in the hospital.

HCPs reported having used OBI and Pre-FS 24 h after myelosuppressive chemotherapy, with 78,33% and 80% doing so, respectively. The least frequent time of administration was at 48–72 h after myelosuppressive chemotherapy, which required the patient’s readmission for administration of pegfilgrastim (see Table 3 for results).

According to our results, 83.3% of professionals use risk scores to evaluate the risk of FN, with the MASCC (Multinational Association of Supportive Care of Cancer) risk index score being the most frequently used (40%), followed by the NCCN (National Comprehensive Cancer Network) risk assessment with 38.33% of professionals reporting its use (n = 23). The proportion of professionals using the ESMO (European Society for Medical Oncology) practice guidelines was equal to the proportion of professionals not using any scale (16.67%).

### Hypothetical clinical cases results

**Fig. 3:**
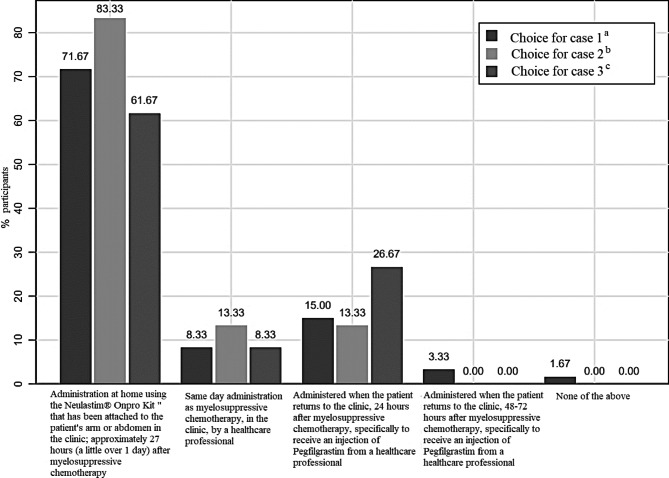
Preferences regarding use of Pegfilgrastim in three hypothetical clinical cases At least 60% of HCP reported a preference for the use of Neulastim OBI for the administration of Pegfilgrastim for all hypothetical cases presented, followed by having the patients return after 24 h of myelosuppressive therapy administration for a Pegfilgrastim injection

Clinical case 1 asked participants to choose a pegfilgrastim administration option solely on their preference with no consideration of a particular patient. Case 2 included a 67-year-old patient with a second recurrence of breast cancer, visceral metastases and COPD; this patient had to travel for 2 h to reach the clinic. Case 3 presented a 29-year-old woman with early-stage breast cancer with surgical resection of the primary tumor and no significant medical history; this patient had to travel for 30 min to reach the clinic. Within the first scenario, the preferred option was the administration of pegfilgrastim at home using OBI with 71.67% (n = 43), which has been attached to the patient’s arm or abdomen in the clinic. The medication is dispensed approximately 27 h after myelosuppressive chemotherapy. This option was also the most common for the second and third scenarios with a frequency of 83.33% (n = 50) and 61.67% (n = 37), respectively. For the first two scenarios, the second most frequent choice was the administration of the drug with the patient re-entering the clinic 24 h after myelosuppressive chemotherapy to receive an injection of pegfilgrastim, with 15% and 13.3%, respectively. The second most popular option in the third scenario was the administration of an injection of pegfilgrastim by a HCP when the patient returned to the clinic, 48–72 h after myelosuppressive chemotherapy, with 26.67%. These results are depicted in Fig. 3.

When analyzing the results for the first, second and third hypothetical clinical cases, it is important to note that 90.7%, 90% and 97.3%, respectively, of participants who chose the option “Administration at home using the OBI” considered it more relevant to prevent the patient from returning to the clinic 24 h after chemotherapy for pegfilgrastim administration. Among the reasons for choosing Administration at home using OBI in the first hypothetical case, the following stand out: patient’s transportation limitations (79.07%; 34/43), releasing of nursing staff to attend to another patient (69.77%; 30/43), and the patient’s fragility (67.44%; 29/43). Furthermore, for the second hypothetical clinical case, the most frequently reported reasons for choosing OBI included patient transport limitations (84%; 42/50), failure to return to the clinic 24 h after chemotherapy (72%; 36/50), and patient fragility (70%; 35/50). The third case displayed a similar pattern as the previous two cases with patient not returning to the clinic 24 h after chemotherapy (81.08%; 30/37), patient’s limitations on transportation (64.86%; 24/37) and releasing of nursing staff to care for another patient (64.86%; 24/37) being the main reasons for HCP’s choices. These results can be found separately for each hypothetical case in Supplementary Tables 1,2,3 and 4, in Appendix 2.

**Fig. 4:**
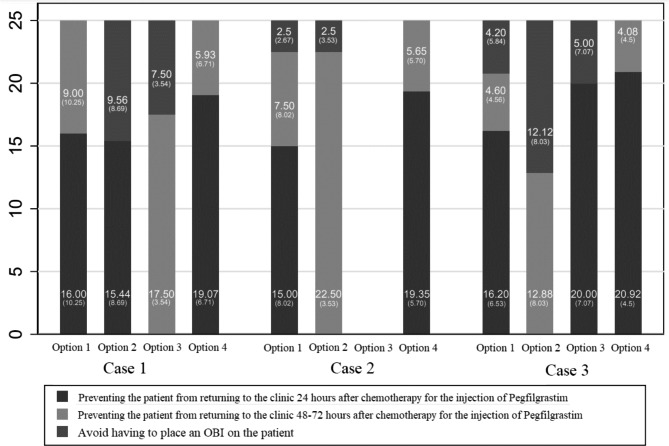
Results for point-allocation exercise between different reasons for choosing a particular Pegfilgrastim administration option The most frequent reason to be considered when choosing a Pegfligrastim administration method is preventing the patient from returning to the center after chemotherapy administration for all hypothetical cases

The overall results for the weighting exercise where professionals were asked to distribute a total of 25 points between a set of reasons that motivate them to choose one pegfilgrastim administration over another is presented in Fig. 4. As it is shown, HCPs prefer to prevent the patient from returning to the clinic 24 h after chemotherapy over other alternatives proposed in all the hypothetical scenarios with average scores that exceed 12.5 points (the middle point of the possible maximum score).

## Discussion

This study aimed to describe HCPs’ preferences regarding the different options for administering pegfilgrastim and how these preferences influence the daily practice of general practitioners, nurses, and hemato-oncology specialists in cancer care settings. So far, we are not aware of any previous studies in Colombia that have evaluated preferences in the use of Neulastim® OBI compared to Pre-FS. Our results indicate that most professionals prefer to avoid having the patient re-enter the care center. However, the patient’s characteristics and ease of transport are some determining factors for those surveyed when deciding the best option for administering pegfilgrastim.

One study estimated, in the United States, the travel burden on patients and informal caregivers to be around 60 million miles and time devoted to such visits at 4.9 million hours or over 12 h per patient treated, annually [11]. Even so, another study comparing access to general (not oncological) healthcare in Colombia and Brazil, found that the mean journey time for patients to reach outpatient secondary level of care is about 62.7 min in Colombia, compared to 36.1 min in Brazil [12]. A long journey time for health reasons could affect working hours, home time and might also imply greater expenses on transportation for both patients and their companions [13].

Colombian patients from rural areas differ very much from patients in Colombian urban areas when evaluating general access to health services and, particularly, to healthcare of chronic and complex diseases such as cancer. Nevertheless, our results elucidate that the viability of recurring visits for each patient is a priority for Colombian HCPs when choosing how to administrate pegfilgrastim without undermining therapeutic efforts. Previous research projects have set the precedent for the detrimental effects of time and economic burden on the likelihood of attendance to follow-up visits for GCSF prophylaxis and optimal care, in general [11, 14].

In our study, around 83% of participants worked at two of the biggest cities in Colombia and one of them is the capital city of the country, so it was expected that such centers had to receive a significant number of patients referred from distal and rural areas. These patients are frequently redirected to the central region of the country were specialists are usually located.

When analyzing the hypothetical cases presented to participants, we found that OBI was the preferred option for all cases, which implied that HCPs chose OBI regardless of patients’ age, cancer stage, complications and even concomitant diseases. Our results suggest that even when a potential patient has to travel a short period of time to reach the clinic, such as 30 min, the treating professional might still prefer to use OBI and prevent the patient from having to return to the clinic 24 h after chemotherapy. It is also interesting to note that HCPs reported having the patient return to the clinic 48–72 h after chemotherapy as the second best option after OBI in clinical case number 3, which presented a young woman with early-stage breast cancer, no major complications and travel time of 30 min to the clinic.

The reasons behind this choice might involve the fact that the patient is less frail than the patient in clinical case 2 and the difference in travel time, which is considerably less for this younger patient. Other reasons reported by HCPs for avoiding re-admission include the release of nursing staff in order to provide care to other patients. Similar findings were already reported by Heubers et al., with OBI as an approach to decrease care burden on healthcare staff while assuring pegfilgrastim administration within the recommended time frame [5]. Furthermore, it is important to mention that most HCPs reported seeing patients who did not belong to the subsidized health insurance regime, which might directly influence their choice since those patients probably have the means of affording the increased cost of OBI prescription thanks to their health insurance scheme.

Results from our study are consistent with the results from Heuber et al. regarding healthcare providers’ main priorities when choosing an option for pegfilgrastim administration. Preventing the patient from returning to the clinic 24 h after chemotherapy for the injection of pegfilgrastim is the most relevant factor when choosing OBI for pegfilgrastim administration and our study suggests that HCPs in Colombia prefer to choose this option regardless of the patients’ clinical condition and travel time to the clinic. The reason for this finding might be related to the workload and limited availability of complex healthcare services to respond to the volume of patients that attend cancer centers at main cities, this situation motivates HCPs to prioritize and optimize physical infrastructure, human resources, and even economic resources. The frequent use of OBI with most patients might be seen by HCPs as a way to relieve such burdens on the health system. These results are consistent with previous studies that have highlighted this benefit when using OBI [15, 16].

A global survey on the delivery of cancer care documented that oncologists from low and middle-income countries see more patients, work more days and are more often on call [17]; additionally, the higher volume of patients they face is associated with less time spent with them. Our findings support previous reports of a perceived need to rearrange oncology staff to meet patients’ needs, possibly due to a very high volume of patients.

According to our results, the majority of physicians we included have chosen Pre-FS administration within the first 24 h after myelosuppressive therapy, as it is the preferred clinically-validated option for time and form of administration of pegfilgrastim, following the international guidelines. However, the proportion of professionals who choose OBI is very close to this, with 48.02% choosing OBI and 51.98% choosing Pre-FS, according to our results. This seems to indicate that both alternatives could be chosen as frequently in daily medical practice. This result is consistent with the general tendency of respondents to avoid having the patient re-enter 48 or 72 h after myelosuppressive therapy and could vary according to each patient’s specific case, as seen in hypothetical clinical situations.

It is important to mention that the group of oncology nurses that participated in this study seemed to prefer OBI in a greater proportion compared to other professionals, such as hematologists or hemato-oncologist. In fact, our results suggest that highly specialized professionals might prefer to comply with international guidelines and choose the traditional form of pegfilgrastim administration through PFS. This result is particularly interesting because the general analysis shows that avoiding the patient from having to return to the clinic so frequently is a priority for most participants, although oncologists and hemato-oncologists still consider PFS their preferred option regardless of patients’ characteristics.

When evaluating our results considering the distribution of cancer centers in the national territory, we find that the greatest concentration of them is in the northern and western region of the country, with the largest cities contributing with the greatest proportion of participants. It is worth noting that Bogotá contributed with 73.3% of the participants, which provides an additional perspective on the presumed flow of patients to specific cities and cancer care centers, making these institutions the most likely healthcare providers for patients referred from the country’s periphery where access to cancer specialists is not an option. By the year 2019 in Colombia, a deficit of between 125 and 179 medical oncologists was estimated to cover the demand for patient care [18]. This consideration is important because it becomes an additional factor to be handled by HCPs when evaluating their own role in the continuum of social support that cancer patients normally require, such as having a relative to accompany them to medical appointments [19]. This kind of support on caregivers’ behalf is important for cancer patients’ mental health and it might be troublesome to accomplish if the HCP instructs the patient to visit the clinic several times.

Finally, it is important to emphasize that approximately 16% of professionals do not report using any risk index for FN in the evaluation of oncological patients. Considering the wide use of risk indexes for clinical decision-making, it would be important to evaluate the performance of the most frequently used tools related to the choice of pegfilgrastim administration option. Moreover, other studies have evaluated the relevance of FN risk assessment in order to avoid unnecessary treatment with pegfilgrastim to optimize patients’ welfare and clinical outcomes [20]. Evaluating these variables exceeds the scope of the present study and is proposed as a potential investigational objective for future studies.

Based on these results, the importance that Colombian professionals have attributed to the heterogeneous characteristics of patients, both in the clinical and social environment, is reflected on the prioritization of the patient’s comfort and optimization of human resources in healthcare when choosing the best option for the administration of pegfilgrastim. One study that analyzed real-world effectiveness of OBI in comparison to PFS reported that there seemed to be no difference in effectiveness in preventing neutropenia [21]; however, our study suggests that the benefits of using OBI reach a greater scope when taking into account particular variables in countries similar to Colombia, where healthcare access and the availability of specialized HCPs is an important issue in patient treatment.

Our results do not differ from those reported previously [1] when evaluating the same variables but show a tendency for HCPs in Colombia to prioritize preventing patients from having to return to the clinic after receiving chemotherapy by choosing the use of OBI more frequently than reported in previous studies [5], these results are probably related to the obstacles faced by HCPs in Colombia, who choose to optimize time and resources to avoid the re-entry of patients, as each case allows. More importantly, our results are consistent with previous findings and suggest that OBI might increase persistence, adherence and compliance to treatment, determining HCPs’ preference towards its use [7].

### Study limitations

As a cross-sectional observational study, results may be limited by the availability of HCPs to participate in the study. Because participants work in specialized centers and because sampling is non-probabilistic, results may not be generalizable. Study participants may not be representative of all patients receiving pegfilgrastim or all physicians prescribing pegfilgrastim, potentially limiting the generalization of study findings. Furthermore, it is important to take into account recall bias, considering that the surveyed participants had to report the most frequent behaviors they had in their professional performance during at least 6 months prior to the survey. The most relevant bias that was taken into account was that of data capture, which was controlled through adequate training of the personnel responsible for administering the survey to the participants and the electronic recording of the data.

## Conclusions

The present study is the first study in Colombia that sought the preferences of HCPs (physicians and nurses) with the use of Neulastim (OBI) compared to Pre-FS for the administration of pegfilgrastim. Our results indicate that most professionals prefer to avoid the patient having to re-enter the care center; however, patient characteristics and ease of transport are a determining factor for respondents when deciding the best option for drug administration. Further research is needed to better understand the patient’s perspective regarding the use of OBI for the administration of pegfilgrastim and the implications of HCPs’ decisions regarding pegfilgrastim administration options on patients’ general satisfaction with care. This study allowed the inclusion of the most representative oncological centers in Colombia and suggests the use of OBI as a good strategy to optimize resources in the context of health care for oncological patients in our country.

## Electronic supplementary material

Below is the link to the electronic supplementary material.

Flowchart 1. Administration on same day after myelosuppressive chemotherapy. Flowchart 2. Administration 24 hours after myelosuppressive chemotherapy. Flowchart 3. Administration 48-72 hours after myelosuppressive chemotherapy. Flowchart 4. Administration 27 hours after myelosuppressive chemotherapy with OBI

Appendix 2: Supplementary Table 1: Description of oncologic management scenarios under Pegfilgrastim use. Supplementary Table 2: Detailed description of the options selected by the health professionals for scenario 1. Supplementary Table 3: Detailed description of the options selected by health professionals for Scenario 2. Supplementary Table 4: Detailed description of the options selected by the health professionals for Scenario 3.

## Data Availability

(data transparency) The datasets used and/or analysed during the current study are available from the corresponding author on reasonable request.

## List of abbreviations

OBI: On-Body Injector
FN: Febrile Neutropenia
GCSF: Granulocyte colony-stimulating factors
HCP: Healthcare professionals
Pre-FS: Pre-filled syringes
ECOG: Eastern Cooperative Oncology Group
MASCC: Multinational Association of Supportive Care of Cancer
NCCN: National Comprehensive Cancer Network
ESMO: European Society for Medical Oncology
