# Supplementary material for: Health professionals’ preferences with the use of pegfilgrastim on-body injector at oncology centers in 8 cities in Colombia
Source: BMC Health Serv Res. 2023 May 23;23:529. doi: 10.1186/s12913-023-09454-z (PMC10207821; doi:10.1186/s12913-023-09454-z)
Supplement: Supplementary file 1 — Flowchart 1. Administration on same day after myelosuppressive chemotherapy. Flowchart 2. Administration 24 hours after myelosuppressive chemotherapy. Flowchart 3. Administration 48-72 hours after myelosuppressive chemotherapy. Flowchart 4. Administration 27 hours after myelosuppressive chemotherapy with OBI [file 12913_2023_9454_MOESM1_ESM.pdf]

Flowchart 1. Administration on same day after myelosuppressive chemotherapy

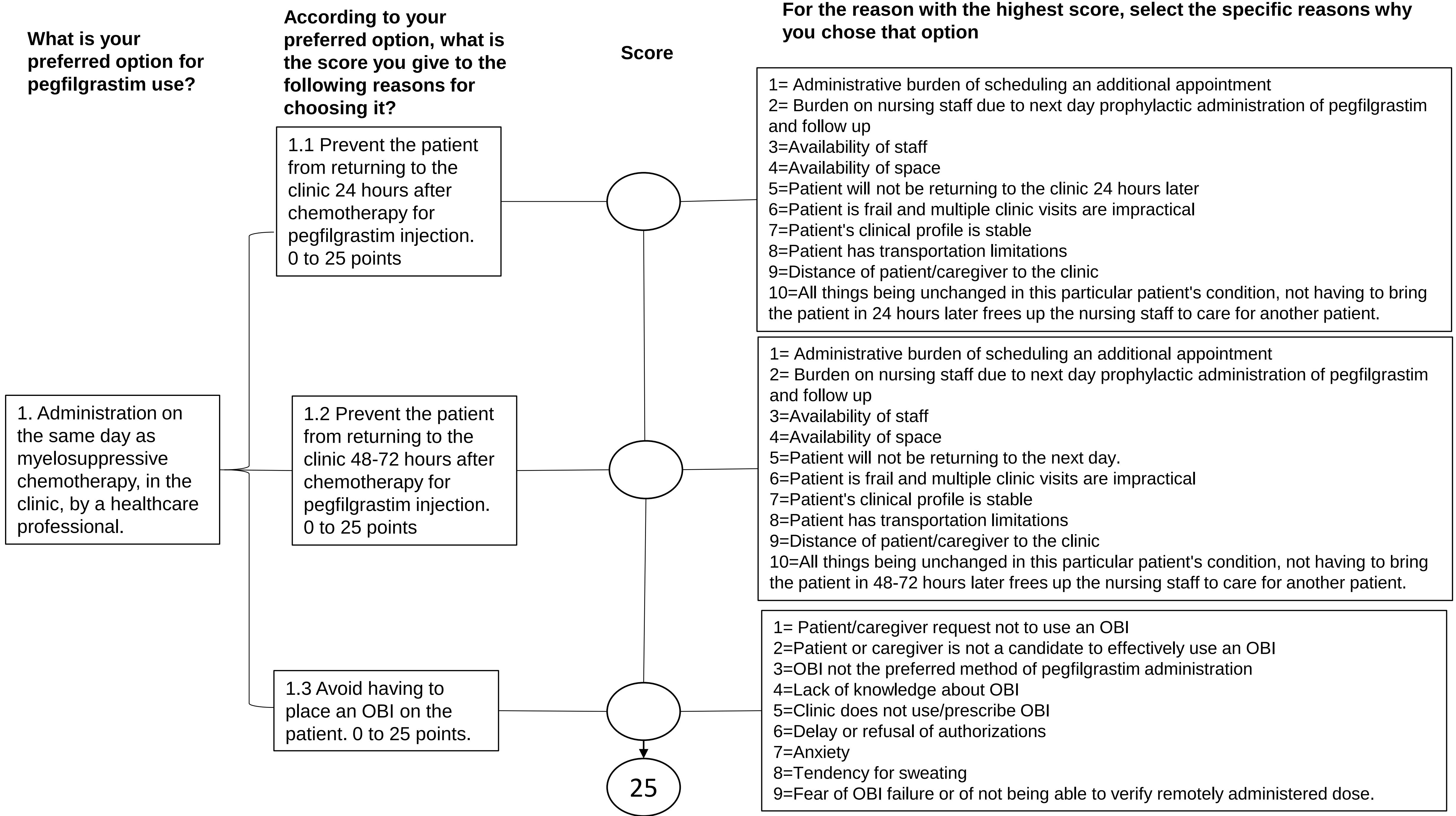

Flowchart 2. Administration 24 hours after myelosuppressive chemotherapy

What is your preferred option for pegfilgrastim use?

According to your preferred option, what is the score you give to the following reasons for choosing it?

Score

For the reason with the highest score, select the specific reasons why you chose that option

2. Administration when the patient returns to the clinic, 24 hours after myelosuppressive chemotherapy, specifically to receive an injection of pegfilgrastim by a health care practitioner

2.1 Prevent the patient from returning to the clinic 48-72 hours after chemotherapy for pegfilgrastim injection. 0 to 25 points

1= Administrative burden of scheduling an additional appointment
2= Burden on nursing staff due to next day prophylactic administration of pegfilgrastim and follow up
3=Availability of staff
4=Availability of space
5=Patient will not be returning to the next day.
6=Patient is frail and multiple clinic visits are impractical
7=Patient's clinical profile is stable
8=Patient has transportation limitations
9=Distance of patient/caregiver to the clinic
10=All things being unchanged in this particular patient's condition, not having to bring the patient in 48-72 hours later frees up the nursing staff to care for another patient.

2.2. Avoid having to place an OBI on the patient 0 to 25 points

1= Patient/caregiver request not to use an OBI
2=Patient or caregiver is not a candidate to effectively use an OBI
3=OBI not the preferred method of pegfilgrastim administration
4=Lack of knowledge about OBI
5=Clinic does not use/prescribe OBI
6=Delay or refusal of authorizations
7=Anxiety
8=Tendency for sweating
9=Fear of OBI failure or of not being able to verify remotely administered dose.

25

Flowchart 3. Administration 48-72 hours after myelosuppressive chemotherapy

What is your preferred option for pegfilgrastim use?

3. Administration when the patient returns to the clinic 48-72 hours after myelosuppressive chemotherapy, specifically to receive an injection of pegfilgrastim by a healthcare professional.

According to your preferred option, what is the score you give to the following reasons for choosing it?

3.1 Prevent the patient from returning to the clinic 24 hours after chemotherapy for pegfilgrastim injection. 0 to 25 points

3.2. Avoid having to place an OBI on the patient 0 to 25 points

Score

25

For the reason with the highest score, select the specific reasons why you chose that option

For 3.1:

1= Administrative burden of scheduling an additional appointment
2= Burden on nursing staff due to next day prophylactic administration of pegfilgrastim and follow up
3=Availability of staff
4=Availability of space
5=Patient will not be returning to the next day.
6=Patient is frail and multiple clinic visits are impractical
7=Patient's clinical profile is stable
8=Patient has transportation limitations
9=Distance of patient/caregiver to the clinic
10=All things being unchanged in this particular patient's condition, not having to bring the patient in 24 hours later frees up the nursing staff to care for another patient.

For 3.2:

1= Patient/caregiver request not to use an OBI
2=Patient or caregiver is not a candidate to effectively use an OBI
3=OBI not the preferred method of pegfilgrastim administration
4=Lack of knowledge about OBI
5=Clinic does not use/prescribe OBI
6=Delay or refusal of authorizations
7=Anxiety
8=Tendency for sweating
9=Fear of OBI failure or of not being able to verify remotely administered dose.

Flowchart 4. Administration 27 hours after myelosuppressive chemotherapy with OBI

What is your preferred option for pegfilgrastim use?

According to your preferred option, what is the score you give to the following reasons for choosing it?

Score

For the reason with the highest score, select the specific reasons why you chose that option

4. Home administration using the Neulasta Onpro Kit, which has been attached to the patient's arm or abdomen in the clinic, approximately 27 hours (a little over 1 day) after myelosuppressive chemotherapy, the injector will beep and blink to indicate that automatic injection of the drug is about to begin.

4.1 Evitar que el paciente regrese a la clínica 24 horas después de la quimioterapia para la inyección de pegfilgrastim. 0 a 25 puntos

1= Administrative burden of scheduling an additional appointment
2= Burden on nursing staff due to next day prophylactic administration of pegfilgrastim and follow up
3=Availability of staff
4=Availability of space
5=Patient will not be returning to the next day.
6=Patient is frail and multiple clinic visits are impractical
7=Patient's clinical profile is stable
8=Patient has transportation limitations
9=Distance of patient/caregiver to the clinic
10=All things being unchanged in this particular patient's condition, not having to bring the patient in 24 hours later frees up the nursing staff to care for another patient.

4.2 Prevent the patient from returning to the clinic 48-72 hours after chemotherapy for pegfilgrastim injection. 0 to 25 points.

1= Administrative burden of scheduling an additional appointment
2= Burden on nursing staff due to next day prophylactic administration of pegfilgrastim and follow up
3=Availability of staff
4=Availability of space
5=Patient will not be returning to the next day.
6=Patient is frail and multiple clinic visits are impractical
7=Patient's clinical profile is stable
8=Patient has transportation limitations
9=Distance of patient/caregiver to the clinic
10=All things being unchanged in this particular patient's condition, not having to bring the patient in 48-72 hours later frees up the nursing staff to care for another patient.

25
