# Supplementary material for: Health professionals’ preferences with the use of pegfilgrastim on-body injector at oncology centers in 8 cities in Colombia
Source: BMC Health Serv Res. 2023 May 23;23:529. doi: 10.1186/s12913-023-09454-z (PMC10207821; doi:10.1186/s12913-023-09454-z)
Supplement: Supplementary file 2 — Appendix 2: Supplementary Table 1: Description of oncologic management scenarios under Pegfilgrastim use. Supplementary Table 2: Detailed description of the options selected by the health professionals for scenario 1. Supplementary Table 3: Detailed description of the options selected by health professionals for Scenario 2. Supplementary Table 4: Detailed description of the options selected by the health professionals for Scenario 3. [file 12913_2023_9454_MOESM2_ESM.docx]

**APPENDIX 2**

**Supplementary Table 1: Description of oncologic management scenarios under Pegfilgrastim use.**

| **Scenario in which the survey will be applied** | **Participants** | |
| --- | --- | --- |
|  | **n=60** | |
|  | **n** | **%** |
|  |  | 60 |
| **Scenario 1 ^a^** |  |  |
|  |  |  |
| **Which of the following is your preferred option for prescribing or administering Pegfilgrastim?** |  |  |
| **Administration when the patient returns to the clinic, 24 hours after myelosuppressive chemotherapy, specifically to receive an injection of pegfilgrastim by a healthcare professional** | 9 | 15,00 |
| **Administration when the patient returns to the clinic, 48-72 hours after myelosuppressive chemotherapy, specifically to receive an injection of pegfilgrastim by a healthcare professional** | 2 | 3,33 |
| **Same day administration as myelosuppressive chemotherapy, in the clinic, by a healthcare professional** | 5 | 8,33 |
| **Home administration using the Neulastim® Onpro Kit " that has been attached to the patient's arm or abdomen in the clinic; approximately 27 hours (slightly more than 1 day) after myelosuppressive chemotherapy.** | 43 | 71,67 |
| **None of the above** | 1 | 1,67 |
|  |  |  |
| **Scenario 2 ^b^** |  |  |
|  |  |  |
| **Which of the following is your preferred option for prescribing or administering Pegfilgrastim?¿** |  |  |
| **Administration when the patient returns to the clinic, 24 hours after myelosuppressive chemotherapy, specifically to receive an injection of pegfilgrastim by a healthcare professional** | 8 | 13,33 |
| **Administration when the patient returns to the clinic, 48-72 hours after myelosuppressive chemotherapy, specifically to receive an injection of pegfilgrastim by a healthcare professional** | 2 | 3,33 |
| **Same day administration as myelosuppressive chemotherapy, in the clinic, by a healthcare professional** | 0 | 0,00 |
| **Home administration using the Neulastim® Onpro Kit " that has been attached to the patient's arm or abdomen in the clinic; approximately 27 hours (slightly more than 1 day) after myelosuppressive chemotherapy** | 50 | 83,33 |
| **None of the above** | 0 | 0,00 |
|  |  |  |
| **Scenario 3 ^c^** |  |  |
|  |  |  |
| **Which of the following is your preferred option for prescribing or administering Pegfilgrastim?** |  |  |
| **Administration when the patient returns to the clinic, 24 hours after myelosuppressive chemotherapy, specifically to receive an injection of pegfilgrastim by a healthcare professional** | 5 | 8,33 |
| **Administration when the patient returns to the clinic, 48-72 hours after myelosuppressive chemotherapy, specifically to receive an injection of pegfilgrastim by a healthcare professional** | 16 | 26,67 |
| **Same day administration as myelosuppressive chemotherapy, in the clinic, by a healthcare professional** | 2 | 3,33 |
| **Home administration using the Neulastim® Onpro Kit " that has been attached to the patient's arm or abdomen in the clinic; approximately 27 hours (slightly more than 1 day) after myelosuppressive chemotherapy** | 37 | 61,67 |
| **None of the above** | 0 | 0,00 |

**^a Scenario based on preference as a healthcare professional for pegfilgrastim use.^**

**^b A 67-year-old woman with chronic obstructive pulmonary disease who presented with a second recurrence of breast cancer involving bone, lung and liver, and an Eastern Cooperative Oncology Group (ECOG) performance status of 2. She was starting a two-drug chemotherapy regimen due to rapidly progressing visceral metastases. She traveled 2 hours to the clinic to receive her chemotherapy infusions.^**

**^c A 29-year-old woman with early-stage breast cancer who was having a first course of dose-dense adjuvant chemotherapy after undergoing limited surgical resection of the primary breast tumor. She had no significant medical history or concomitant illnesses and had an ECOG performance status of 0. She traveled 30 minutes to the clinic to receive her chemotherapy infusions.^**

**Supplementary Table 2: Detailed description of the options selected by the health professionals for scenario 1**

| **Variables** | **Participants** | |
| --- | --- | --- |
|  | **n=60** | |
|  | **n** | **%** |
|  |  | 60 |
| **SCENARIO 1** |  |  |
|  |  |  |
| ***Option: Same day administration as myelosuppressive chemotherapy, in the clinic, by a health care professional.*** | 5 | 8,33 |
| **Point allocation** |  |  |
| **Prevent the patient from returning to the clinic 24 hours after chemotherapy for pegfilgrastim injection. ^e^** | 16 (10,25) | 15 (15 - 25) |
| **Prevent the patient from returning to the clinic 48-72 hours after chemotherapy for pegfilgrastim injection. ^e^** | 9 (10,25) | 10 (0 - 10) |
|  |  |  |
|  |  |  |
| **What were the reasons for choosing this option? ^a^** |  |  |
| **Administrative burden of scheduling an additional appointment** | 3 | 60 |
| **Burden on nursing staff due to next-day prophylactic pegfilgrastim administration and follow up** | 3 | 60 |
| **Staff availability** | 3 | 60 |
| **Space availability** | 3 | 60 |
| **The patient will not return to the clinic 24 hours later** | 3 | 60 |
| **The patient is fragile and visits to several clinics are not practical** | 4 | 80 |
| **The patient's clinical profile is stable** | 2 | 40 |
| **Patient has transportation limitations** | 4 | 80 |
| **Distance from patient / care to clinic** | 4 | 80 |
| **If everything remains unchanged in this particular patient's condition; not having to bring the patient in 24 hours later frees up the nursing staff to care for another patient.** | 1 | 20 |
|  |  |  |
| ***Option: Administration when the patient returns to the clinic, 24 hours after myelosuppressive chemotherapy, specifically to receive an injection of pegfilgrastim by a healthcare professional.*** | 9 | 15,00 |
| **Point allocation** |  |  |
| **Prevent the patient from returning to the clinic 24 hours after chemotherapy for pegfilgrastim injection. ^e^** | 15,44 (8,69) | 15 (5 - 24) |
| **Avoiding the need to place an OBI on the patient ^e^** | 9,56 (8,69) | 10 (1 - 20) |
|  |  |  |
|  |  |  |
| **What were the reasons for choosing this option? ^b^** |  |  |
| **Administrative burden of scheduling an additional appointment** | 0 | 0,00 |
| **Burden on nursing staff due to next-day prophylactic pegfilgrastim administration and follow up** | 0 | 0,00 |
| **Staff availability** | 1 | 11,11 |
| **Space availability** | 2 | 22,22 |
| **The patient will not return to the clinic 24 hours later** | 0 | 0,00 |
| **The patient is fragile and visits to several clinics are not practical** | 2 | 22,22 |
| **The patient's clinical profile is stable** | 1 | 11,11 |
| **Patient has transportation limitations** | 4 | 44,44 |
| **Distance from patient / care to clinic** | 5 | 55,56 |
| **If everything remains unchanged in this particular patient's condition; not having to bring the patient in 24 hours later frees up the nursing staff to care for another patient.** | 0 | 0,00 |
|  |  |  |
| ***Option: Administration when the patient returns to the clinic, 48-72 hours after myelosuppressive chemotherapy, specifically to receive an injection of pegfilgrastim by a healthcare professional.*** | 2 | 3,33 |
| **Point allocation** |  |  |
| **Prevent the patient from returning to the clinic 48-72 hours after chemotherapy for pegfilgrastim injection ^e^** | 17,5 (3,54) | 17,5 (15 - 20) |
| **Avoiding the need to place an OBI on the patient ^e^** | 7,5 (3,54) | 7,5 (5 - 10) |
|  |  |  |
|  |  |  |
| **What were the reasons for choosing this option? ^c^** |  |  |
| **Administrative burden of scheduling an additional appointment** | 1 | 50,00 |
| **Burden on nursing staff due to next-day prophylactic pegfilgrastim administration and follow up** | 0 | 0,00 |
| **Staff availability** | 0 | 0,00 |
| **Space availability** | 0 | 0,00 |
| **The patient will not return to the clinic 24 hours later** | 1 | 50,00 |
| **The patient is fragile and visits to several clinics are not practical** | 0 | 0,00 |
| **The patient's clinical profile is stable** | 0 | 0,00 |
| **Patient has transportation limitations** | 1 | 50,00 |
| **Distance from patient / care to clinic** | 0 | 0,00 |
| **If everything remains unchanged in this particular patient's condition; not having to bring the patient in 24 hours later frees up the nursing staff to care for another patient.** | 1 | 50,00 |
|  |  |  |
| ***Option: Home administration using the Neulastim® Onpro Kit " that has been attached to the patient's arm or abdomen in the clinic; approximately 27 hours (slightly more than 1 day) after myelosuppressive chemotherapy.*** | 43 | 71,67 |
| **Point allocation** |  |  |
| **Prevent the patient from returning to the clinic 24 hours after chemotherapy for pegfilgrastim injection ^e^** | 19,07 (6,71) | 20 (15 - 25) |
| **Avoiding the need to place an OBI on the patient ^e^** | 5,93 (6,71) | 5 (0 - 10) |
|  |  |  |
|  |  |  |
| **What were the reasons for choosing this option? ^d^** |  |  |
| **Administrative burden of scheduling an additional appointment** | 8 | 18,60 |
| **Burden on nursing staff due to next-day prophylactic pegfilgrastim administration and follow up** | 13 | 30,23 |
| **Staff availability** | 8 | 18,60 |
| **Space availability** | 9 | 20,93 |
| **The patient will not return to the clinic 24 hours later** | 25 | 58,14 |
| **The patient is fragile and visits to several clinics are not practical** | 29 | 67,44 |
| **The patient's clinical profile is stable** | 22 | 51,16 |
| **Patient has transportation limitations** | 34 | 79,07 |
| **Distance from patient / care to clinic** | 0 | 0,00 |
| **If everything remains unchanged in this particular patient's condition; not having to bring the patient in 24 hours later frees up the nursing staff to care for another patient.** | 30 | 69,77 |

**^a based on n=5^**

**^b based on n=9^**

**^c based on n=2^**

**^d based on n=43^**

**^e Reported values in means (SD) and medians (p25-p75)^**

**Supplementary Table 3: Detailed description of the options selected by health professionals for Scenario 2**

| **Variables** | **Participants** | |
| --- | --- | --- |
|  | **n=60** | |
|  | **n** | **%** |
|  |  | 60 |
| **SCENARIO 2** |  |  |
|  |  |  |
| ***Option: Same day administration as myelosuppressive chemotherapy, in the clinic, by a health care professional.*** | 8 | 13,33 |
| **Point allocation** |  |  |
| **Preventing the patient from returning to the clinic 24 hours after chemotherapy for pegfilgrastim injection ^d^** | 15 (8,02) | 15 (12,5 - 20) |
| **Avoid having the patient return to the clinic 48-72 hours after chemotherapy for pegfilgrastim injection ^d^** | 7,5 (8,02) | 5 (2,5 - 10) |
| **Avoid having to place an OBI in the patient ^d^** | 2,5 (2,67) | 2,5 (0 - 2) |
|  |  |  |
|  |  |  |
| **What were the reasons for choosing this option? ^a^** |  |  |
| **Administrative burden of scheduling an additional appointment** | 2 | 25 |
| **Burden on nursing staff due to next-day prophylactic pegfilgrastim administration and follow up** | 1 | 12,5 |
| **Staff availability** | 1 | 12,5 |
| **Space availability** | 1 | 12,5 |
| **Patient will not return to the clinic 24 hours later** | 0 | 0 |
| **Patient is frail and multiple clinic visits are impractical** | 6 | 75 |
| **Patient's clinical profile is stable** | 1 | 12,5 |
| **Patient has transportation limitations** | 6 | 75 |
| **Distance from patient/care to clinic** | 4 | 50 |
| **All things being unchanged in this particular patient's condition; not having to bring the patient back 24 hours later frees up nursing staff to care for another patient** | 3 | 37,5 |
|  |  |  |
| ***Option: Administration when the patient returns to the clinic, 24 hours after myelosuppressive chemotherapy, specifically to receive an injection of pegfilgrastim by a healthcare professional.*** | 2 | 3,33 |
| **Point allocation** |  |  |
| **Avoid having the patient return to the clinic 24 hours after chemotherapy for pegfilgrastim injection ^d^** | 22,5 (3,53) | 22,5 (20 - 25) |
| **Avoid having to place an OBI on the patient ^d^** | 2,5 (3,53) | 2,5 (0 - 5) |
|  |  |  |
| **Which of the above options obtained the highest score? ^b^** |  |  |
| **Avoid having the patient return to the clinic 48-72 hours after chemotherapy for pegfilgrastim injection.** | 2 | 100,00 |
| **Avoid having to place an OBI on the patient** | 0 | 0,00 |
|  |  |  |
| **What were the reasons for choosing this option? ^b^** |  |  |
| **Administrative burden of scheduling an additional appointment** | 0 | 0,00 |
| **Burden on nursing staff due to next-day prophylactic pegfilgrastim administration and follow up** | 1 | 50,00 |
| **Staff availability** | 0 | 0,00 |
| **Space availability** | 0 | 0,00 |
| **Patient will not return to the clinic 24 hours later** | 0 | 0,00 |
| **Patient is frail and multiple clinic visits are impractical** | 0 | 0,00 |
| **Patient's clinical profile is stable** | 0 | 0,00 |
| **Patient has transportation limitations** | 1 | 50,00 |
| **Distance from patient/care to clinic** | 1 | 50,00 |
| **All things being unchanged in this particular patient's condition; not having to bring the patient back 24 hours later frees up nursing staff to care for another patient** | 0 | 0,00 |
|  |  |  |
| ***Option: Administration when the patient returns to the clinic, 48-72 hours after myelosuppressive chemotherapy, specifically to receive an injection of pegfilgrastim by a healthcare professional.*** | 0 | 0,00 |
|  |  |  |
| ***Option: Home administration using the Neulastim® Onpro Kit " that has been attached to the patient's arm or abdomen in the clinic; approximately 27 hours (slightly more than 1 day) after myelosuppressive chemotherapy.*** | 50 | 83,33 |
| **Point allocation** |  |  |
| **Avoid having the patient return to the clinic 24 hours after chemotherapy for pegfilgrastim injection ^d^** | 19,35 (5,70) | 20 (15 - 25) |
| **Avoid having to place an OBI in the patient ^d^** | 5,65 (5,70) | 5 (0 - 10) |
|  |  |  |
|  |  |  |
| **What were the reasons for choosing this option? ^c^** |  |  |
| **Administrative burden of scheduling an additional appointment** | 12 | 24,00 |
| **Burden on nursing staff due to next-day prophylactic pegfilgrastim administration and follow up** | 12 | 24,00 |
| **Staff availability** | 9 | 18,00 |
| **Space availability** | 11 | 22,00 |
| **Patient will not return to the clinic 24 hours later** | 36 | 72,00 |
| **Patient is frail and multiple clinic visits are impractical** | 35 | 70,00 |
| **Patient's clinical profile is stable** | 19 | 38,00 |
| **Patient has transportation limitations** | 42 | 84,00 |
| **Distance from patient/care to clinic** | 0 | 0,00 |
| **All things being unchanged in this particular patient's condition; not having to bring the patient back 24 hours later frees up nursing staff to care for another patient** | 33 | 66,00 |

**^a based on n=8^**

**^b based on n=2^**

**^c based on n=50^**

**^d Reported values in means (SD) and medians (p25-p75)^**

**Supplementary Table 4: Detailed description of the options selected by the health professionals for Scenario 3**

| **Variables** | **Participants** | |
| --- | --- | --- |
|  | **n=60** | |
|  | **n** | **%** |
|  |  | 60 |
| **SCENARIO 3** |  |  |
|  |  |  |
| ***Option: Same day administration as myelosuppressive chemotherapy, in the clinic, by a health care professional.*** | 5 | 8,33 |
| **Point allocation** |  |  |
| **Prevent patient from returning to the clinic 24 hours after chemotherapy for pegfilgrastim injection^e^** | 16,2 (6,53) | 15 (13 - 20) |
| **Avoid having the patient return to the clinic 48-72 hours after chemotherapy for pegfilgrastim injection ^e^** | 4,6 (4,56) | 5 (0 - 8) |
| **Avoid having to place an OBI in the patient ^e^** | 4,2 (5,84) | 0 (0 - 9) |
|  |  |  |
| **Which of the above options obtained the highest score? ^a^** |  |  |
| **Prevent the patient from returning to the clinic 24 hours after chemotherapy for pegfilgrastim injection.** | 4 | 80 |
| **Prevent the patient from returning to the clinic 48-72 hours after chemotherapy for pegfilgrastim injection.** | 1 | 20 |
|  |  |  |
| **What were the reasons for choosing this option? ^a^** |  |  |
| **Administrative burden of scheduling an additional appointment** | 1 | 20 |
| **Burden on nursing staff due to next-day prophylactic pegfilgrastim administration and follow up** | 1 | 20 |
| **Staff availability** | 0 | 0 |
| **Space availability** | 1 | 20 |
| **Patient will not return to the clinic 24 hours later** | 1 | 20 |
| **Patient is frail and multiple clinic visits are impractical** | 0 | 0 |
| **Patient's clinical profile is stable** | 1 | 20 |
| **Patient has transportation limitations** | 1 | 20 |
| **Distance from patient/care to clinic** | 2 | 40 |
| **All things being unchanged in this particular patient's condition; not having to bring the patient back 24 hours later frees up nursing staff to care for another patient** | 2 | 40 |
|  |  |  |
| ***Option: Administration when the patient returns to the clinic, 48-72 hours after myelosuppressive chemotherapy, specifically to receive a pegfilgrastim injection by a healthcare professional*** | 16 | 26,67 |
| **Point allocation** |  |  |
| **Avoid having the patient return to the clinic 48-72 hours after chemotherapy for pegfilgrastim injection ^e^** | 12,88 (8,03) | 11 (7,5 - 20) |
| **Avoid having to place an OBI in the patient ^e^** | 12,12 (8,03) | 14 (5 - 17,5) |
|  |  |  |
|  |  |  |
| **What were the reasons for choosing this option? ^b^** |  |  |
| **Administrative burden of scheduling an additional appointment** | 1 | 6,25 |
| **Burden on nursing staff due to next-day prophylactic pegfilgrastim administration and follow up** | 1 | 6,25 |
| **Staff availability** | 1 | 6,25 |
| **Space availability** | 1 | 6,25 |
| **Patient will not return to the clinic 24 hours later** | 0 | 0,00 |
| **Patient is frail and multiple clinic visits are impractical** | 2 | 12,50 |
| **Patient's clinical profile is stable** | 2 | 12,50 |
| **Patient has transportation limitations** | 6 | 37,50 |
| **Distance from patient/care to clinic** | 5 | 31,25 |
| **All things being unchanged in this particular patient's condition; not having to bring the patient back 24 hours later frees up nursing staff to care for another patient** | 0 | 0,00 |
|  |  |  |
| ***Option: Administration when the patient returns to the clinic, 24 hours after myelosuppressive chemotherapy, specifically to receive an injection of pegfilgrastim by a healthcare professional.*** | 2 | 3,33 |
| **Point allocation** |  |  |
| **Avoid having the patient return to the clinic 24 hours after chemotherapy for pegfilgrastim injection ^e^** | 20 (7,07) | 20 (15 - 25) |
| **Avoid having to place an OBI in the patient ^e^** | 5 (7,07) | 5 (0 - 10) |
|  |  |  |
| **Which of the above options obtained the highest score? ^c^** |  |  |
| **Avoid having the patient return to the clinic 24 hours after chemotherapy for pegfilgrastim injection.** | 2 | 100,00 |
| **Avoid having to place an OBI on the patient** | 0 | 0,00 |
|  |  |  |
| **What were the reasons for choosing this option? ^c^** |  |  |
| **Administrative burden of scheduling an additional appointment** | 1 | 50,00 |
| **Burden on nursing staff due to next-day prophylactic pegfilgrastim administration and follow up** | 0 | 0,00 |
| **Staff availability** | 1 | 50,00 |
| **Space availability** | 1 | 50,00 |
| **Patient will not return to the clinic 24 hours later** | 1 | 50,00 |
| **Patient is frail and multiple clinic visits are impractical** | 1 | 50,00 |
| **Patient's clinical profile is stable** | 1 | 50,00 |
| **Patient has transportation limitations** | 0 | 0,00 |
| **Distance from patient/care to clinic** | 1 | 50,00 |
| **All things being unchanged in this particular patient's condition; not having to bring the patient back 24 hours later frees up nursing staff to care for another patient** | 1 | 50,00 |
|  |  |  |
| ***Option: Home administration using the Neulastim® Onpro Kit " that has been attached to the patient's arm or abdomen in the clinic; approximately 27 hours (slightly more than 1 day) after myelosuppressive chemotherapy.*** | 37 | 61,67 |
| **Point allocation** |  |  |
| **Prevent patient from returning to the clinic 24 hours after chemotherapy for pegfilgrastim injection ^e^** | 20,92 (4,5) | 20 (18 - 25) |
| **Prevent the patient from returning to the clinic 48-72 hours after chemotherapy for pegfilgrastim injection ^e^** | 4,08 (4,5) | 5 (0 - 7) |
|  |  |  |
|  |  |  |
| **What were the reasons for choosing this option? ^d^** |  |  |
| **Administrative burden of scheduling an additional appointment** | 9 | 24,32 |
| **Burden on nursing staff due to next-day prophylactic pegfilgrastim administration and follow up** | 10 | 27,03 |
| **Staff availability** | 9 | 24,32 |
| **Space availability** | 6 | 16,22 |
| **Patient will not return to the clinic 24 hours later** | 30 | 81,08 |
| **Patient is frail and multiple clinic visits are impractical** | 19 | 51,35 |
| **Patient's clinical profile is stable** | 14 | 37,84 |
| **Patient has transportation limitations** | 24 | 64,86 |
| **Distance from patient/care to clinic** | 0 | 0,00 |
| **All things being unchanged in this particular patient's condition; not having to bring the patient back 24 hours later frees up nursing staff to care for another patient** | 24 | 64,86 |

**^a based on n=5^**

**^b based on n=16^**

**^c based on n=2^**

**^d based on n=37^**

**^e Reported values in means (SD) and medians (p25-p75)^**
